# Immunostimulatory Activities of Theobromine on Macrophages via the Activation of MAPK and NF-κB Signaling Pathways

**DOI:** 10.3390/cimb44090289

**Published:** 2022-09-12

**Authors:** Hee-Weon Lee, In-Wook Choi, Sang Keun Ha

**Affiliations:** 1 Korea Food Research Institute, 245, Nongsaengmyeong-ro, Iseo-myeon, Wanju-gun 55365, Korea; 2 School of Pharmacy, Sungkyunkwan University, Suwon 16419, Korea; 3 Division of Food Biotechnology, University of Science and Technology, Daejeon 35408, Korea

**Keywords:** theobromine, inflammation, macrophage, MAPK, NF-κB

## Abstract

Theobromine is mainly found in plant foods, such as tea; the primary source of theobromine is the seeds of the Theobroma cacao tree. Theobromine is an alkaloid belonging to the methylxanthine class of drugs, and it is similar to theophylline and caffeine. Theobromine is known for its efficacy and role in health and disorder prevention. We evaluated the effects of theobromine on macrophage function, including the phosphorylation of mitogen-activated protein kinases (MAPKs) and nuclear factor-kappa B (NF-κB). Theobromine significantly stimulated the production of nitric oxide (NO) and prostaglandin E2 through immune responses, which relate to the increased expression of inducible nitric oxide synthase and cyclooxygenase-2. Additionally, theobromine increased the production of inflammatory cytokines, including tumor necrosis factor-α and interleukin-6 in macrophages. Additionally, theobromine induced the translocation and activity of NF-κB in a concentration-dependent manner. Consistent with these results, the phosphorylation level of MAPKs was increased in theobromine-stimulated macrophages. Collectively, these data revealed that theobromine acts as an immune response stimulator via the NF-κB and MAPKs signaling pathways. Thus, theobromine might have protective effects against inflammatory disorders.

## 1. Introduction

It has been reported that many natural medicinal compounds, acting as immune regulatory factors, can stimulate immune responses. Theobromine (3,7-Dimethylxanthine), like caffeine, belongs to the xanthine group of alkaloids and acts as a stimulant in the body [1]. Theobromine is mainly sourced from the seed of the cacao tree, but this chemical occurs naturally in many plant foods [2]. It acts as an adenosine receptor antagonist, food ingredient, plant metabolite, vasodilator, and bronchodilator. Previously, it was used to treat angina and hypertension. Recently, several studies have examined the association of theobromine with obesity, diabetes, and inflammatory diseases [3].

In order to protect our body from sources of infection, inflammation is produced by immune responses. Several types of macrophages are present in the tissues of each organ, as well as circulate white blood cells, including monocytes and neutrophils, to counter infection in human body. These innate immune cells recognize pathogen invasion or cellular damage with intracellular or surface-expressed pattern-recognition receptors [4]. Chronic inflammation is associated with the cause of various diseases, including cancer, rheumatoid arthritis, type 2 diabetes, and various metabolic syndromes [5].

Inflammation is a key cause of many pathological conditions, such as obesity [6], cancer, arthritis, atopy, and many other diseases, so modulation of inflammation is an important target for disease control [7]. It acts as a key regulator in the pathogenesis of many diseases, such as metabolic disorders, cancer, and bacterial infection. Inflammatory responses are controlled by several different immune cells including macrophages, which are known to play essential roles in host defense, inflammatory responses, and immune responses [8]. Macrophages also have a pivotal function in inflammatory disorders related to the over-expression of pro-inflammatory cytokines (such as TNF-α, IL-6, IL-1β, and IL-18) and mediators of inflammatory response (including NO, PGE2, MAPKs, and NF-κB) [9]. Therefore, inflammatory diseases could be treated by modulating the factors associated with macrophages. Macrophages are key inflammatory response regulators, and toll-like receptors (TLRs) are the most characteristic inducers of acute inflammation. When macrophages are stimulated by inflammatory factors, such as lipopolysaccharides (LPS), oxidase stress, and viruses, numerous gene changes are induced that cause an immune response. Thus, macrophages play critical roles not only in innate immunity but also in adaptive immunity. Stimulated macrophages release several inflammatory cytokines and nitric oxide (NO), which directly induce inflammatory activity [10]. These responses also cause a variety of genetic modifications to regulate the signaling system [11,12].

Several studies have demonstrated that NF-kB contributes to the anti-inflammatory process by modulating inflammatory cell development and regulating gene expression through an antioxidant response. The transcription factor NF-kB modulates the immune response by regulating inflammatory factors. Various stimuli factors, including pathogen-related molecular patterns, stimulate cell surface receptors such as the members of the TLR family to initiate signal cascades, resulting in the activation of NF-kB [13]. NF-kB is an essential transcription gene that regulates various immune responses, including the release of cytokines and expression of various inflammatory mediators [14]. Indeed, many studies of various diseases have indicated a major role for the signaling pathway of NF-kB in the development of inflammation-related metabolic diseases [15].

In macrophages, NF-kB is localized to the cytoplasm by binding with IκBα, an NF-κB inhibitor. p65 is presented to the cytoplasm and binds to an inhibitor protein known as IκB. However, when IκB is phosphorylated by stimulation, NF-κB is released, and p65 translocates to the nucleus and stimulates the expression of several genes, including genes encoding cytokines [15]. Through this immune response cascade, inflammatory cytokines, including TNF-α and IL-6, are generated. In addition, these pro-inflammatory cytokines accumulate in organisms, leading to the development of several disorders [16,17].

The objective of this study was to investigate the immunoregulatory activity of theobromine in immune responses and determine the involved molecular mechanisms. Specifically, our findings indicated that theobromine stimulates the immune responses of macrophages through the MAPK/NF-κB signaling pathway [18,19]. We found that theobromine induces the activation of p65 and MAPKs, as well as the subsequent increase in the expression of several mediators involved in the inflammatory responses and pro-inflammatory cytokines in macrophages. Our findings show that theobromine stimulates an immunostimulatory effect through the MAPK/NF-κB signaling pathway in macrophages. Taken together, these data indicated that theobromine could have significant effects on the modulation of innate immune response and potential therapeutic value in preventing inflammation and inflammatory diseases.

## 2. Materials and Methods

### 2.1. Chemical Reagents and Antibodies

Theobromine was purchased from Sigma-Aldrich (St. Louis, MO, USA). Dulbecco’s Modified Eagle’s Medium (DMEM), and fetal bovine serum (FBS) were obtained from Gibco (BRL, Carlsbad, CA, USA). Most chemicals, including MAPK and NF-κB inhibitors, were obtained from Sigma Chemical Co., unless otherwise stated. Theobromine was dissolved in distilled water and diluted at the indicated concentrations (0.1–25 μg/mL). Antibodies against target molecules were purchased from Cell Signaling (Danvers, MA, USA) and Santa Cruz Biotechnology (Santa Cruz, CA, USA).

### 2.2. Cell Culture

The murine macrophage cell line RAW 264.7 was obtained from ATCC (Rockville, MD, USA), and the RAW 264.7 cells were cultured in DMEM (Lonza, Basel, Switzerland) supplemented with 2 mM of l-glutamine, 100 IU/mL of penicillin, 100 μg/mL of streptomycin, and 10% heat-inactivated FBS. The cells were incubated at 37 °C in a fully humidified incubator containing 5% CO_2_ and were subcultured twice weekly. RAW 264.7 cells were pretreated for 2 h with sinigrin, followed by treatment with LPS (1 μg/mL) for various prespecified times. The cells were harvested by centrifugation at 13,000× *g* for 3 min at 4 °C, and the pellets were lysed by radioimmunoprecipitation assay buffer (RIPA buffer).

### 2.3. Assessment of Cell Proliferation

This method uses the principle that mitochondria reduce 3-(4,5-dimethylthiazol-2-yl)-2,5-diphenyltetrazolium bromide (MTT) to insoluble formazan. Cell viability was determined over a period of 2 h, using the MTT quantitative colorimetric assay to detect the mitochondrial activity in living cells. Using the dehydrogenase action, the soluble substrate MTT tetrazolium penetrates into the living cell and is reduced to formazan, which becomes purple in color, in the mitochondria. The formazan crystal is dissolved in an organic solvent such as dimethyl sulfoxide (DMSO). The absorbance of the formazan dye was measured using an enzyme-linked immunosorbent assay (ELISA) reader (Molecular Devices, Carlsbad, CA, USA) at 540 nm.

### 2.4. Measurement of NO Production

NO production was measured by determining the reduction in the stable nitrite form using the Griess reagent system. Cells were incubated with various concentrations of theobromine (0.1–25 μg/mL) for 24 h in a 5% CO_2_ incubator. After stimulation of theobromine for 1 day, supernatant was collected and reacted with Griess reagent at room temperature. The absorbance was measured at 540 nm using an ELISA reader, and the measured value is expressed as percentage in terms of NO production rate. The concentration of NO_2_^−^, which is indicative of the amount of NO production, was calculated from a NaNO_2_ standard curve.

### 2.5. Determination of Inflammatory Cytokine Production

Macrophages were dispensed at 5 × 10^5^ cells/well in 96-well plates. Cells were treated with different concentration of theobromine (0.1–25 μg/mL) for 24 h. Then, the supernatant was measured using an ELISA kit (R&D system, Minneapolis, MN, USA), in accordance with the instructions of the manufacturer. The level of each cytokine was determined using a standard curve obtained from the reaction of a standard substance.

### 2.6. Immunoblotting Assay

RAW 264.7 macrophages were cultured into 60 mm cell culture plates at a density of 2 × 10^6^ cells/well. Macrophages were then treated with various concentrations of theobromine. After treatment, the cultured cells were rinsed with PBS and suspended in a homogenizer lysis buffer. The supernatant was collected after centrifugation for 15 min at 15,000× *g* and 4 °C. The protein concentration was determined using a DC protein assay kit (Bio-Rad, Hercules, CA, USA). Proteins were separated by 6–15% polyacrylamide gel and transferred to the nitrocellulose (NC) membrane. The protein-transferred membrane was blocked in 0.05%/Tris-buffered saline containing 5% skim milk powder for 1 h and then reacted with primary and secondary antibodies. The blots were developed using an enhanced chemiluminescence kit. The expression of each protein was quantified using image J software. The measured value is expressed as the average value of three repeated experiments, and the experiment was performed in a concentration range that did not cause toxicity.

### 2.7. Quantitative Real Time-PCR

RAW 264.7 macrophages were seeded at a density of 5 × 10^5^ cells/well in 6-well plates. Macrophages were incubated in the presence or absence of theobromine (0.1–25 µg/mL) for 24 h. After theobromine treatment, total RNA was isolated from cultured cells using an RNA extraction kit (Kusatsu, St. Shiga, Japan), in accordance with the instructions of the manufacturer, and used for cDNA synthesis. Then, 10 μL of SYBR green premix (Bio-Rad), 8 μL of sterile water, and 1 μL each of forward and reverse primer were mixed to adjust the total volume to 20 μL. Fluorescence was measured at each cycle. Gene expression was normalized using glyceraldehyde-3-phosphate dehydrogenase (GAPDH) as a reference gene. The RT-PCR primer sequences used to analyze the expression of cytokines are listed in Table 1.

### 2.8. Cytosol and Nuclear Extract Preparation

RAW 264.7 macrophages were cultured into 60 mm cell culture plates at a density of 2 × 10^6^ cells/well. Macrophages were then treated with various concentrations of theobromine. After treatment, the cultured cells were pelleted by centrifugation and then rinsed 2–3 times in iced PBS. Pelleted cells were resuspended in buffer A and incubated on ice for 1 h with vortexing. Subsequently, cytosol extract in the supernatant was obtained by centrifugation. The nuclear protein pelleted by centrifugation was suspended in Buffer C and incubated on ice for 1 h, with vortexing every 15 min. The supernatant containing the nuclear protein extract was separated by centrifugation and transferred to a new centrifuge tube to obtain pure nuclear protein. Separated cytoplasmic and nuclear proteins were stored at −20 °C.

### 2.9. Transfection and Luciferase Reporter Activity Assay

RAW 264.7 macrophages were cultured in 60 mm cell culture plates at a density of 2 × 10^6^ cells/well. Macrophages were transfected with pGL3-p65 and pCMV-β-galactosidase (pCMV-β-gal) using lipofectamine RNA iMAX (Invitrogen, Waltham, MA, USA), in accordance with the instructions of the manufacturer. Following transfection for 6 h, cells were incubated in the presence or absence of theobromine (0.1–25 µg/mL) for 24 h. Stimulated cells were washed with iced PBS and then suspended in lysis buffer (Promega Corporation, Madison, WI, USA). Luciferase activity was measured in the lysed cells using a luciferase assay kit (Promega Corporation, Madison, WI, USA), in accordance with the instructions of the manufacturer. The luciferase activity was normalized to β-gal activity. The relative activity is presented as the percentage activity of the control with standard deviation.

### 2.10. Statistical Analysis

For each experiment, experiments were performed in triplicate, and the results are reported as means ± SEM. Comparisons of means between theobromine-treated cells and untreated control cells were made using Student’s *t*-test and ANOVA using the SigmaPlot 10.0 software. Significant values are indicated by an asterisk (* *p* < 0.05).

## 3. Results

### 3.1. Effect of Theobromine on the Proliferation of RAW 264.7 Macrophages

**Figure 1:**
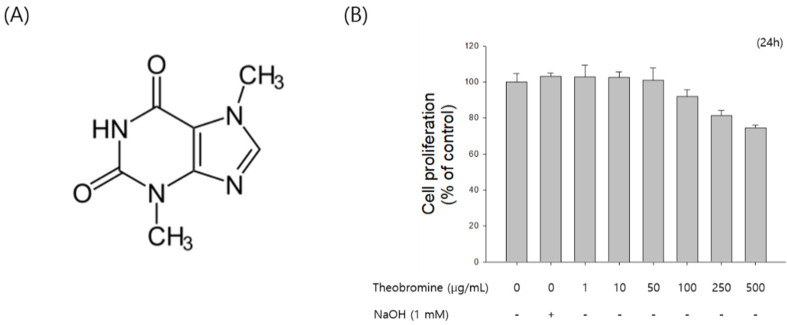
Effect of theobromine on the proliferation of RAW 264.7 macrophages. (**A**) Chemical structure of theobromine. (**B**) RAW 264.7 macrophages were exposed to various concentrations of theobromine for 24 h. Cell viability was examined using a MTT assay. Data are presented as means ± SEM of quintuplicates from a representative experiment.

First, the effect of theobromine on the proliferation of RAW 264.7 cells was assessed using the MTT assay. RAW 264.7 macrophages were treated with the indicated concentrations (1–500 μg/mL) of theobromine for 24 h. Theobromine did not show any cytotoxic effect at concentrations from 1 to 50 μg/mL (Figure 1B). However, theobromine cytotoxicity was observed at higher concentrations. Moreover, cell proliferation was affected by 7.2% at 100 μg/mL. Thus, concentrations of less than 50 μg/mL were used in all subsequent experiments.

### 3.2. Effect of Theobromine on the Production of NO and Prostaglandin E2 (PGE_2_) in RAW 264.7 Macrophages

**Figure 2:**
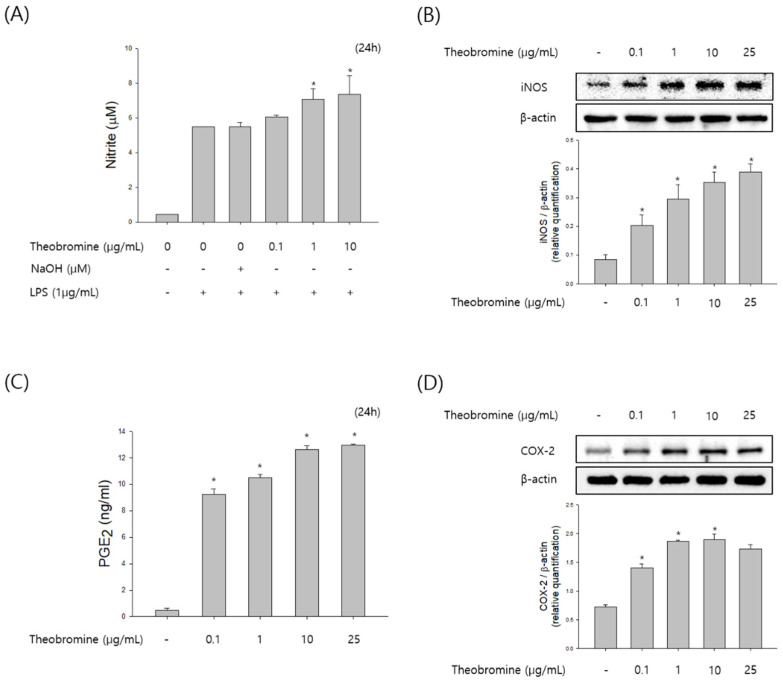
Effect of theobromine on the production of NO and PGE_2_ in RAW 264.7 macrophages. Macrophages were treated with different concentrations of theobromine for 24 h. (**A**) Nitrite oxide in the incubated supernatant was determined using the Griess reagent. (**C**) The production of PGE_2_ in the culture media was investigated by a ELISA assay kit. The protein expression levels of (**B**) iNOS and (**D**) COX-2 were examined by Western blot assay. β-actin protein levels were used as an internal control. Results are presented as means ± SEM of quintuplicates from a representative experiment. * *p* < 0.05, compared with the untreated group.

To examine the effect of theobromine on immunostimulatory activity, we evaluated the production of NO and PGE_2_ in theobromine treated RAW 264.7 macrophages. RAW 264.7 macrophages were incubated with 0.1–25 μg/mL of theobromine for 24 h. After incubation with theobromine, the levels of NO and PGE_2_ in the supernatant were detected by the Griess reagent and ELISA assay. As shown in Figure 2A,B, treatment with theobromine stimulated the production of NO and PGE_2_ in a concentration-dependent manner. We also investigated the effect of theobromine on inducible nitric oxide synthase (iNOS) and cyclooxygenase-2 (COX-2) levels in theobromine cultured cells. The expression levels of iNOS and COX-2 were markedly stimulated by theobromine in a concentration-dependent manner (Figure 2C,D). These data suggest that theobromine possesses immunomodulation effect by regulating the function of macrophages.

### 3.3. Effect of Theobromine on the Secretion of Inflammatory Cytokines by RAW 264.7 Macrophages

**Figure 3:**
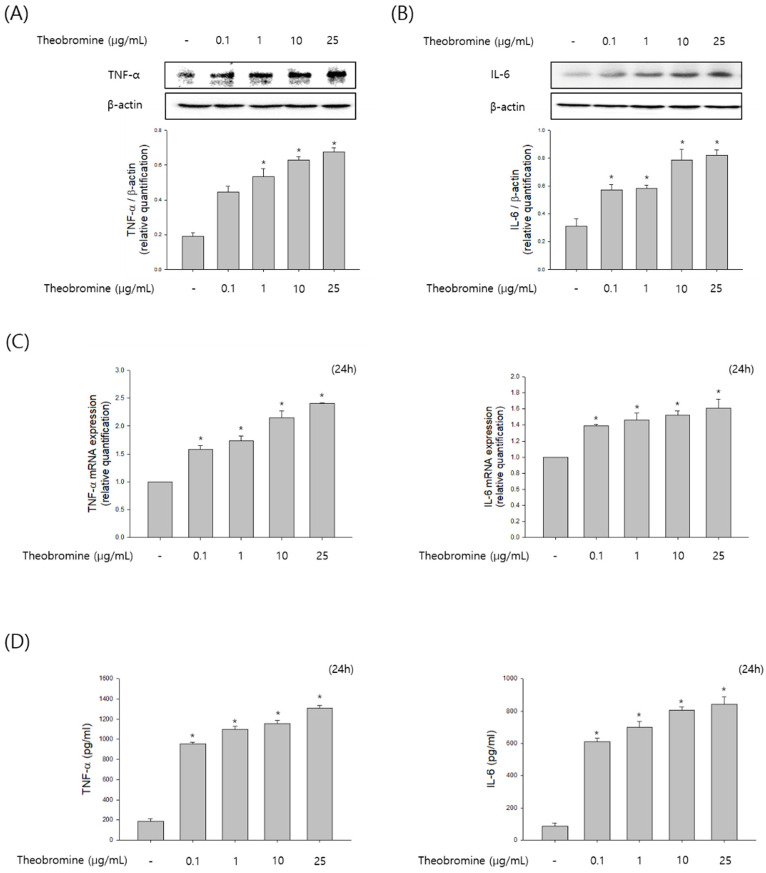
Effect of theobromine on the secretion of inflammatory cytokines in RAW 264.7 macrophages. RAW 264.7 macrophages were treated with the indicated concentrations of theobromine for 24 h. (**A**,**B**) The protein expressions of TNF-α and IL-6 were determined using Western blot assay. (**C**) The mRNA levels of TNF-α and IL-6 were determined by RT-PCR. (**D**) The secretion amounts of TNF-α and IL-6 were measured using an ELISA kit. Results are presented as means ± SEM of quintuplicates from a representative experiment. * *p* < 0.05, compared with the untreated group.

To investigate whether theobromine regulates the level of cytokines production by activating macrophages, RAW 264.7 macrophages were incubated with theobromine at 1–25 μg/mL for 24 h. We elucidated the protein and mRNA expression of inflammatory cytokines by Western blot and qRT-PCR analyses. As shown in Figure 3A–C, treatment with theobromine increased the protein and mRNA levels of TNF-α and IL-6 in RAW 264.7 macrophages. To validate these results, the level of cytokines released in the supernatants was measured by the ELISA assay. These data indicated that theobromine remarkably up-regulated the secretion of TNF-α and IL-6 in a concentration-dependent manner compared to the control (Figure 3D). Taken together, these results indicate that theobromine exerts its effects by stimulating the production of cytokines and, thus, the immune responses.

### 3.4. Effect of Theobromine on the Translocation and Activation of NF-κB in RAW 264.7 Macrophages

**Figure 4:**
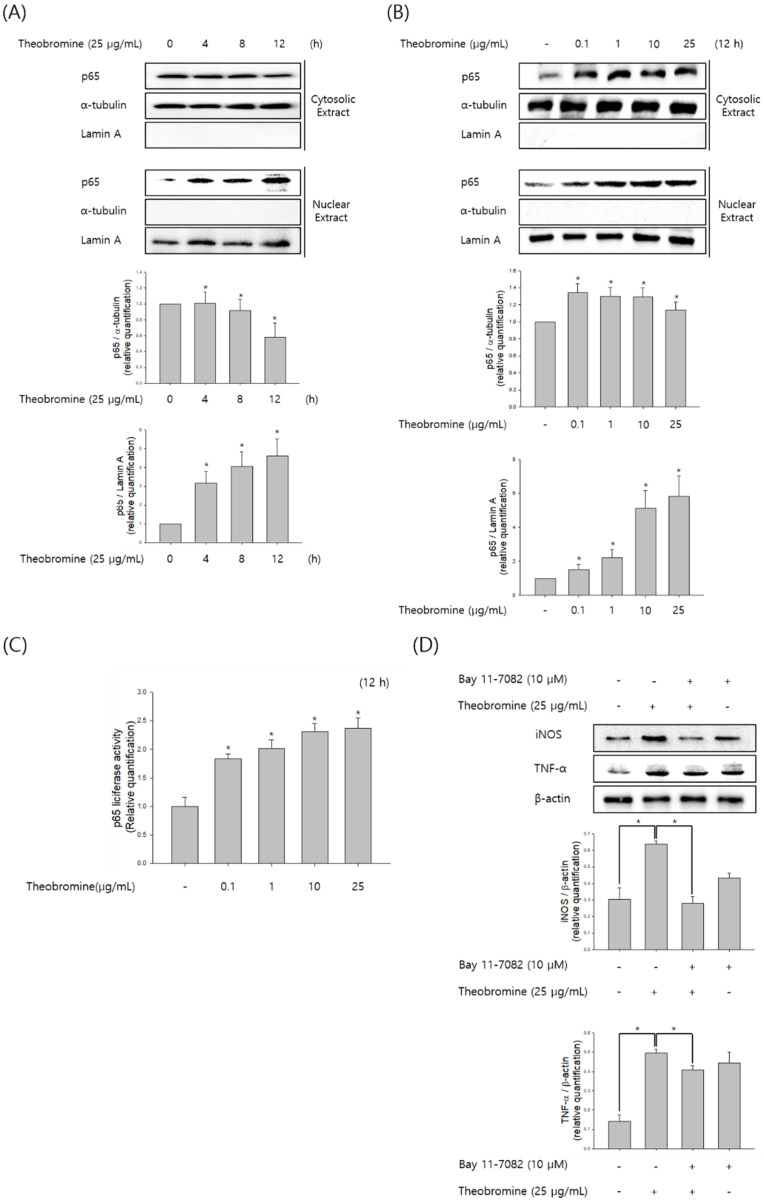
Effect of theobromine on the activation and translocation of NF-κB in RAW 264.7 macrophages. Macrophages were stimulated with theobromine for 4 h. (**A**,**B**) The level of p65 was determined at the indicated times or concentrations by Western blot assay to analyze the translocation of NF-κB. (**C**) Macrophages were transfected with a pGL3-p65-Luc reporter plasmid and β-galactosidase and incubated with various concentrations of theobromine for 4 h. NF-κB activation was determined by luminometry. (**D**) Macrophages were incubated with the NF-κB inhibitor Bay 11-7082 for 2 h and then stimulated with theobromine for 4 h. Whole cell lysates were examined using Western blot assay. β-actin protein levels were used as an internal control. Results are shown as means ± SEM of quintuplicates from a representative experiment. * *p* < 0.05, compared with the untreated group.

NF-κB is an essential transcription factor and regulates inflammatory cytokine and NO production during inflammatory responses. To determine whether theobromine increases NF-κB levels, RAW 264.7 macrophages were incubated with theobromine for the indicated time and concentration, and then the nuclear and cytoplasmic levels of NF-κB p65 subunit were determined by Western blot analysis. Western blot analysis revealed that theobromine increased NF-κB p65 translocation from the cytosol to the nucleus (Figure 4A,B). As shown in Figure 4C, we also examined the effect of theobromine on NF-κB activity using a pGL3-NF-κB-Luc reporter plasmid and pCMV-β-gal. Stimulation of the macrophages with theobromine resulted in a 2.3-fold increase in luciferase activity. Furthermore, we investigated the effect of the NF-κB inhibitor Bay 11-7082 in theobromine-stimulated macrophages. The increased levels of NO and pro-inflammatory cytokines that were induced by theobromine treatment were significantly suppressed by incubation with Bay 11-7082 (Figure 4D). Collectively, these results suggest that theobromine can increase the activation of NF-κB, leading to inflammatory responses through the regulation of inflammatory mediators.

### 3.5. Effect of Theobromine on the Phosphorylation of MAPKs in RAW 264.7 Macrophages

**Figure 5:**
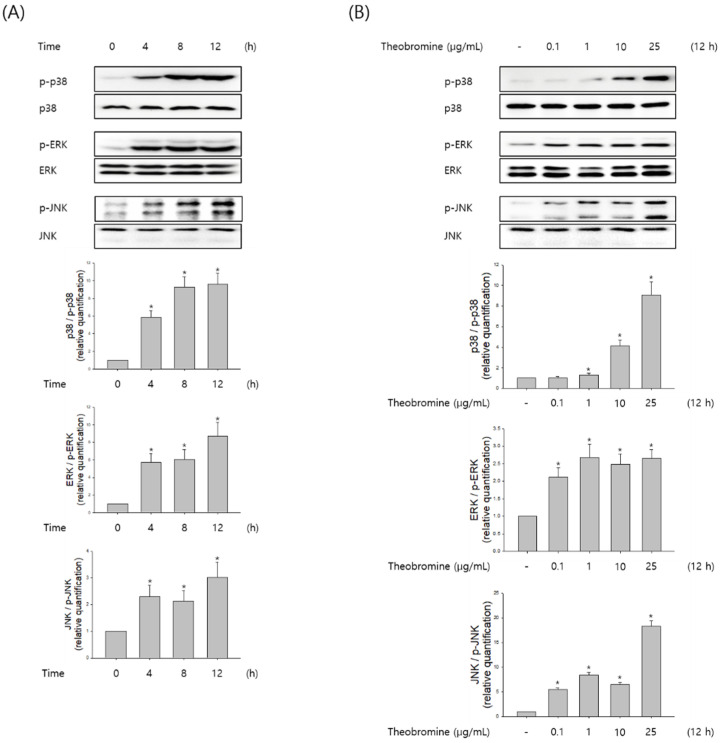

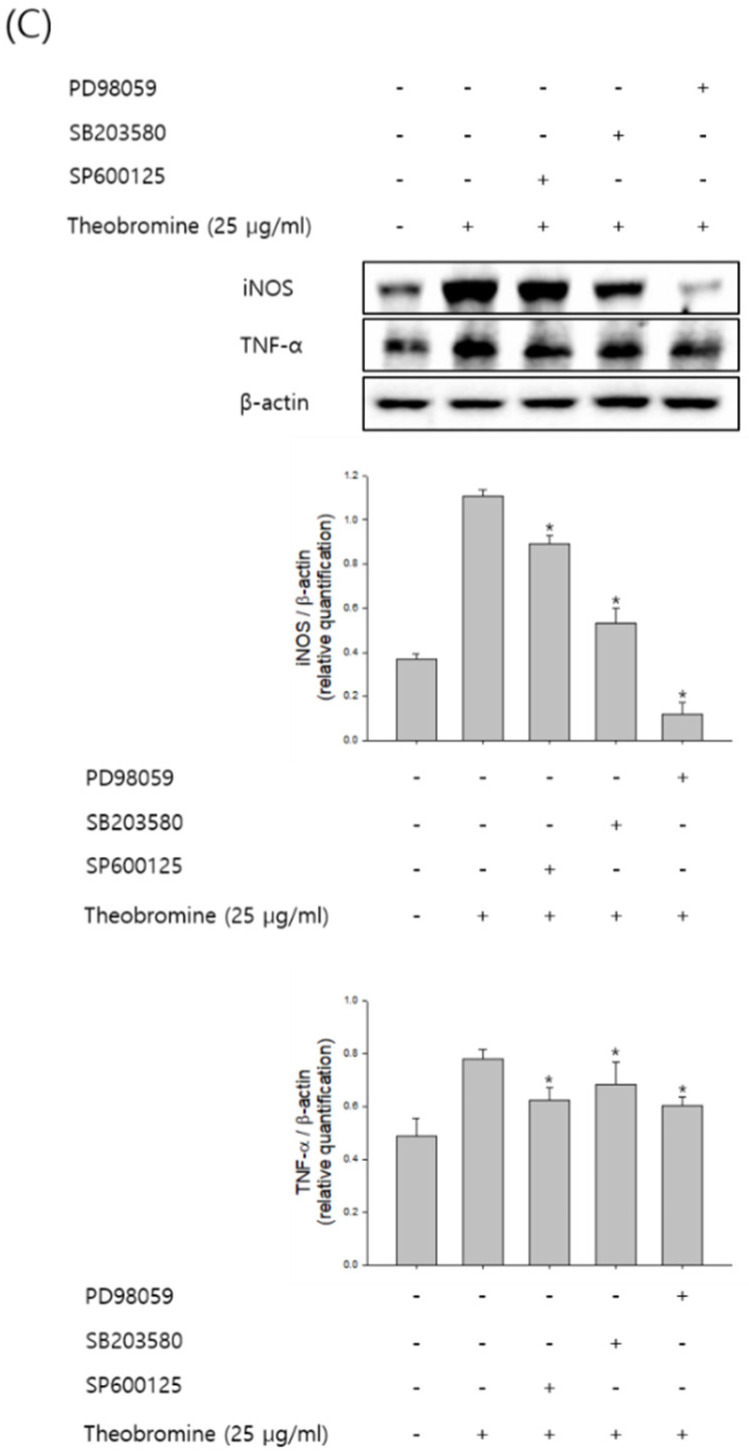
Effect of theobromine on the phosphorylation of MAPKs in RAW 264.7 macrophages. Macrophages were treated with theobromine for the various times and at the indicated concentrations. (**A**,**B**) The phosphorylation level of MAPKs was detected at the indicated times or concentrations by Western blot assay to confirm the MAPKs phosphorylation. (**C**) Macrophages were treated with theobromine for 1 h in the presence or absence of the ERK1/2 inhibitor PD98059 (10 μM), the JNK inhibitor SP600125 (20 μM), and the p38 MAPK inhibitor SB203580 (20 μM). Whole cell lysates were analyzed by Western blot assay. β-actin protein levels were used as an internal control. Results are shown as means ± SEM of quintuplicates from a representative experiment. * *p* < 0.05, compared with the untreated group.

MAPKs are known to play an important role in the regulation of several inflammatory mediators in macrophages. Therefore, macrophages were stimulated with theobromine at the indicated time and concentration, and then the phosphorylation levels of ERK1/2, JNK, and p38 were evaluated by Western blot analysis. Theobromine induced the phosphorylation of MAPKs in a concentration-dependent manner compared to the control, after a 12 h treatment (Figure 5A,B). To determine whether the MAPK signaling pathway is associated with NO and pro-inflammatory cytokines in theobromine-activated macrophages, we evaluated the effects of the ERK1/2 inhibitor PD98059, the JNK inhibitor SP600125, and the p38 MAPK inhibitor SB203580 on the expression of NO and TNF-α. As shown in Figure 5C, macrophages were incubated with the inhibitors for 2 h, after being stimulated with theobromine for 24 h. Treatment with theobromine increased the phosphorylation of MAPK. However, when RAW 264.7 macrophages were treated with specific MAPK inhibitors, the theobromine-induced expression of NO and TNF-α was almost diminished. All these observations suggest that theobromine could stimulate the inflammatory mediators through the MAPKs signaling pathways.

## 4. Discussion

In the present study, we elucidated the ability and molecular mechanisms of theobromine to enhance the production of inflammatory mediators and activation of MAPK and NF-kB in macrophages. We first confirmed that theobromine induced the inflammatory activity of RAW 264.7 macrophages. Indeed, theobromine has been demonstrated to exhibit strong macrophage activity. Therefore, we investigated its biological effects on the expression and activation of inflammatory mediators in inflammatory responses. The results showed that theobromine induced NO and PGE_2_ production and the activity of pro-inflammatory cytokines TNF-α and IL-6. Thus, we identified the inflammatory mediators that activate inflammatory factors in RAW 264.7 macrophages. Collectively, it was confirmed that theobromine triggered inflammatory activity through the activation of MAPK and NF-kB in RAW 264.7 macrophages. Although theobromine has been reported to be an inflammatory activity, its exact mechanism in the production of inflammatory mediators is still unknown [20,21]. To the best of our knowledge, this is the first report describing the mechanisms involved in the immunostimulatory effects of theobromine, which enhance the production of pro-inflammatory mediators via down-regulation of p38 and JNK phosphorylation and NF-κB activation in macrophages.

Inflammation is an immune response involving various signaling pathways. Inflammatory response is a necessary mechanism for responding to many diseases in our body. It also causes several diseases such as rheumatoid arthritis, asthma, arteriosclerosis, and cancer [5,22]. Inflammatory reactions occur for a variety of reasons. Appropriate inflammation is beneficial to humans. MAPKs and NF-κB are known to play critical roles in the production of NO and PGE_2_ as well as the release of pro-inflammatory cytokines including TNF-α, IL-6, IL-1β, and IL-18 [23,24]. For this reason, we examined the regulation of inflammatory mediators and cytokines by theobromine in macrophages. Furthermore, we investigated the effect of theobromine on the activation of MAPKs and NF-κB in inflammatory responses. Therefore, we determined how theobromine affects the inflammatory response through MAPKs and NF-κB in macrophages. Macrophages, which play an essential role in immunity, regulate various inflammatory responses stimulated by external antigens. In this process, macrophages also up-regulate the production of various inflammatory mediators to participate in the inflammatory responses [25]. The production and activation of inflammatory cytokines among various inflammatory factors arising from an inflammatory reaction are considered to be particularly important in inflammatory mechanisms [26]. In addition, an appropriate NO increase enhances the immune response. Furthermore, PGE2 is also involved in the fundamental mediation of the inflammation signaling activated by the production of COX-2 in stimulated macrophages [27]. Therefore, diminution of NO and PGE_2_ production may be effective in the regulation of inflammatory reactions. Here, theobromine significantly increased PGE_2_ levels through the activation of COX-2 and inflammatory cytokines [16]. In addition, theobromine specifically promotes the production of NO by increasing the expression of iNOS in stimulated macrophages. These results indicate that theobromine activated the inflammation-related factors produced by macrophages. Accordingly, this suggests that theobromine activates the mechanisms of immune response through the inflammatory response [28,29].

Much research has suggested that the regulation of the MAPK and NF-kB pathways is a fundamental factor to the activation of inflammatory responses. Therefore, regulation of the immune response through these factors is an important process. MAPK, which responds to several external stimuli such as LPS and internal disorders, is activated in macrophages, and activated MAPK leads to the activation of important mediators [30]. The current study data indicated that theobromine increased phosphorylation by regulating MAPKs, indicating that the MAPKs signaling pathway is related to the inflammatory processes that are mediated by theobromine [19,31]. In macrophages, the expression of the COX gene is a critical factor for producing PGE_2_ and is important for the inflammatory response. The transcription factor NF-kB is also a key factor for inducing an inflammatory signaling through various mechanisms in the inflammatory responses. In unstimulated cells, the p65/p50 heterodimer interacts with IκBα in the cytoplasm [30,32]. When the cells are stimulated, NF-kB and IκBα are separated, allowing nuclear translocation of p65, which subsequently activates the expression of the inflammatory genes [7,15]. Theobromine induces IκBα degradation and p65 translocation into the nucleus in stimulated macrophages. This suggests that theobromine exerts inflammatory activity by causing phosphorylation of IκBα and p65 translocation. Inflammatory disorders are related to the overexpression of inflammatory cytokines and abnormal immune responses to external stimuli. Therefore, proper expression of cytokines maintains an appropriate inflammatory response in the body [23]. During immune responses, the production of pro-inflammatory cytokines, including TNF-α and IL-6, is enhanced in macrophages, and these cytokines are also released by other immune-related cells [14]. TNF-α and IL-6 have essential roles in the regulation of inflammatory mechanisms and responses. These inflammatory cytokines are secreted by a variety of cells, including macrophages, through the regulation of the MAPK and NF-κB signaling pathways. Based on these findings, theobromine seems to regulate the release of cytokines through these inflammatory mechanisms [33].

In this study, theobromine increased the immune activity of macrophages. In addition, theobromine induced the expression of iNOS and COX-2 in a concentration-dependent manner, thereby inducing the production of inflammatory cytokines. The MAPK and NF-κB signaling pathways are associated with the secretion of TNF-α, IL-16, and inflammatory mediators by macrophages [4]. Furthermore, theobromine can trigger the production of several inflammatory factors and cytokines through the regulation of the MAPK and NF-κB signaling pathways. Consistent with our results, many papers have already demonstrated these results. In addition to these facts, our study confirmed that theobromine influences the interaction between macrophages and inflammation as a stimulating factor. In summary, our data confirmed that theobromine generates macrophage inflammatory factors and induces immune activity in macrophages.

## 5. Conclusions

In summary, the collective results demonstrated that theobromine increased the activity of inflammatory mediators in stimulated macrophages, providing a possible molecular mechanism for the immune activity of theobromine. The immune-enhancing effect of theobromine was mediated through the activation of the MAPK and NF-κB signaling pathways in macrophages. Our results revealed that theobromine is able to increase the production of inflammatory mediators via the p38, JNK, and NF-κB signaling pathways. In addition, we also found that theobromine increased the production of NO by up-regulating the expression of iNOS. In addition, AGE increased the expression of COX-2, resulting in increased production of PGE2. Taken together, these data revealed the mechanisms through which theobromine influences the inflammatory activity of macrophages, providing insights into the approach of using theobromine as a possible treatment for inflammatory-related diseases.

## Figures and Tables

**Table 1:** Primer sequences and real-time PCR conditions.

Gene	Forward Primer (5′→3′)	Reverse Primer (5′→3′)
TNF-α	CCC TCA CAC TCA GAT CAT CTT CT	GCT ACG ACG TGG GCT ACA G
IL-6	CCA CGG CCT TCC CTA CTT C	TTG GGA GTG GTA TCC TCT GTG A
GAPDH	TGC ATC CTG CAC CAC CAA	TCC ACG ATG CCA AAG TTG TC

## Data Availability

Data is contained within the article.
